# Incorporating media data into a model of infectious disease transmission

**DOI:** 10.1371/journal.pone.0197646

**Published:** 2019-02-04

**Authors:** Louis Kim, Shannon M. Fast, Natasha Markuzon

**Affiliations:** Information and Cognition Division, The Charles Stark Draper Laboratory, Cambridge, MA, United States of America; Columbia University, UNITED STATES

## Abstract

Understanding the effect of media on disease spread can help improve epidemic forecasting and uncover preventive measures to slow the spread of disease. Most previously introduced models have approximated media effect through disease incidence, making media influence dependent on the size of epidemic. We propose an alternative approach, which relies on real data about disease coverage in the news, allowing us to model low incidence/high interest diseases, such as SARS, Ebola or H1N1. We introduce a network-based model, in which disease is transmitted through local interactions between individuals and the probability of transmission is affected by media coverage. We assume that media attention increases self-protection (e.g. hand washing and compliance with social distancing), which, in turn, decreases disease model. We apply the model to the case of H1N1 transmission in Mexico City in 2009 and show how media influence—measured by the time series of the weekly count of news articles published on the outbreak—helps to explain the observed transmission dynamics. We show that incorporating the media attention based on the observed media coverage of the outbreak better estimates the disease dynamics from what would be predicted by using media function that approximate the media impact using the number of cases and rate of spread. Finally, we apply the model to a typical influenza season in Washington, DC and estimate how the transmission pattern would have changed given different levels of media coverage.

## Introduction

Disease transmission takes place in a dynamic social environment, wherein individual health decisions are guided by cultural norms, peer influence, and media influence. Recognizing the importance of individuals’ actions in preventing the spread of infection, researchers are beginning to explore mathematical models that incorporate such actions [1, 2]. These models have been used to inform strategies to control the spread of disease [3] and to quantify the role of individual protective actions in controlling several outbreaks, including the 2014 Ebola outbreak in West Africa [4], the 2003 SARS outbreak in Hong Kong [5] and the 2009 H1N1 outbreak in Central Mexico [6].

A number of models have linked media communication about a disease to protective action [7–11]. These models postulate that media influence increases with the number of infected people [7–9], or with both the number of infected people and the rate of change [10, 11]. Models typically assume that media influence reduces the effective transmission rate, slowing the spread of disease. The susceptible-infected-recovered (SIR) framework used to evaluate the effect of media on disease transmission can be described by the following set of equations:

$$\dot{S}=-f(I,\frac{dI}{dt},p_{1},\ldots ,p_{k})\beta SI$$ (1)

$$\dot{I}=f(I,\frac{dI}{dt},p_{1},\ldots ,p_{k})\beta SI-\gamma I$$ (2)

$$\dot{R}=\gamma I$$ (3)

N=S+I+R (4)

In effect, the population (of size *N*) is divided into three groups: those susceptible to infection (*S*), those currently infected (*I*), and those who have recovered from infection (*R*). The effect of media, *f*, is an increasing function of the number of infected individuals and/or the rate of change in the number of infected, controlled by a set of parameters, *p*_1_…*p*_*k*_. The media function slows the rate of transmission of the disease when the number of cases is high or when the prevalence of disease is increasing rapidly, creating interesting disease spread dynamics, such as multi-wave outbreaks [8, 9]. It is not clear, however, that the media function formalization suggested by the models adequately reflects actual media influence [12]. The choice of media function critically influences the shape of the disease spread [12], making accurate parameterization of media crucial. Models are just beginning to consider ways to incorporate data on actual media coverage [13].

Here, we introduce a network-based model, in which disease spread is transmitted through local interactions between individuals, and the probability of transmission is affected by media coverage. We assume that media attention to the outbreak increases self-protection (e.g. hand washing, face mask usage, and compliance with social distancing), which, in turn, decreases disease spread. We model media signal as a function of the actual number of articles published about the disease. Therefore, the media signal in our model is independent of the size of the outbreak. The proposed model is an extension of our previous model, ALARM [14], which attempted to quantify the level of social reaction to disease, but which did not take the role of media into account in the disease transmission process. The key observation that drove the formulation of the ALARM model was that public reaction to a disease is frequently disproportionate to the number of cases. Some outbreaks with very few cases trigger outsized public alarm—for example, the handful of cases of Ebola in the US—while larger and more deadly outbreaks—such as the annual outbreaks of seasonal influenza—generate little interest. Factors such as the novelty of the disease in the area and its clinical severity play a role in shaping the public reaction. By incorporating actual media data into models of disease transmission, we can begin to account for those factors in the transmission dynamics and evaluate their significance in the disease transmission process.

We apply the model to the case of 2009 influenza A(H1N1) transmission in Mexico City and show how media influence—measured by the time series of weekly count of news articles published on the outbreak—helps to explain the observed transmission dynamics. We show that the observed media coverage of the outbreak differs substantially from what would be predicted by approximate media functions. Finally, we apply the model to a typical influenza season in Washington, DC and estimate how the transmission pattern would have changed given different levels of media coverage, showing that under typical conditions media has limited effect on the spread of disease.

## Methods

We introduce a model of disease spread that incorporates media attention and influence in disease spread dynamics. In the model, we explicitly quantify media influence, which leads to a reduction in the per-contact probability of disease transmission.

### SIR model formulation with media function incorporating media coverage data

Our proposed SIR model incorporating media effects differs from earlier formulations [7–11] in two primary ways. First, the media effect is formulated as a function of the actual number of articles published about the disease and is therefore independent of the size of the outbreak. Secondly, instead of the standard deterministic approach with homogenous mixing, we opt to develop a network-based model that operates in discrete time. Since the number of media articles published is discontinuous with respect to time, the use of a discrete time approach is helpful for model parameterization.

#### Disease transmission

We implement a susceptible-infected-recovered (SIR) model [15], adapted for network-based modeling [16, 17]. Each individual, *i*’s, disease state at time *t* is represented by $X_{t}^{i}\in \left\{S,I,R\right\}$, where *S* = susceptible, *I* = infected, and *R* = recovered. Infection is transmitted through pair-wise contact with infected neighbors on the disease network. At time *t*, an infected individual infects each of her susceptible neighbors, independently, with probability *p*_*t*_. Thus, if $X_{t}^{i}=I$, $X_{t}^{j}=S$, and i and j are neighbors on the disease network, then:

$$\begin{array}{c} X_{t+1}^{j}=\{\begin{array}{cc} I & \text{with}\;\text{probability}\;p_{t}\\ S & \text{with}\;\text{probability}\;1-p_{t}. \end{array} \end{array}$$ (5)

Following infection, individuals recover after *T*_*R*_ time periods. Therefore, if $X_{t-1}^{i}=S$ and $X_{t}^{i}=I$, then:

$$\begin{array}{c} X_{t}^{i}=\cdots =X_{t+T_{R}-1}^{i}=I\;\text{and}\;X_{t+T_{R}}^{i}=R. \end{array}$$ (6)

When vaccine is available, we implement an imperfect vaccine, with a delay of *d* time units before becoming effective. For influenza, the delay before full immunity is approximately two weeks [18]. Vaccines are distributed randomly among the susceptible population, according to the estimated number of vaccines administered during the week. Let *η* be the vaccine efficacy. Then, if susceptible individual, *i*, is vaccinated at time *t*:

$$\begin{array}{c} X_{t+d}^{j}=\{\begin{array}{cc} R & \text{with}\;\text{probability}\;\eta \\ S & \text{with}\;\text{probability}\;1-\eta . \end{array} \end{array}$$ (7)

It has been shown that formulating imperfect vaccination as an all-or-nothing effect with probability of vaccination success equal to the vaccine efficacy correctly estimates the direct effectiveness of the vaccine [19].

#### Media function

We assume that media attention to the outbreak increases self-protective behavior (e.g. hand washing, face mask usage, and compliance with social distancing) [20–23], which, in turn, decreases disease transmission [24, 25]. Specifically, we define the per-contact transmission probability at time *t* as the product of a baseline transmission probability, *p*_0_, and the media function, *g*:

$$\begin{array}{c} p_{t}=g(M_{0},\ldots ,M_{t},\ldots M_{T},\alpha ,\lambda )p_{0}, \end{array}$$ (8)

where *M*_*t*_ is the number of news articles published at time *t*, and *α* and λ are parameters, controlling the change in transmission probability resulting from media influence and the relative weight of recent and prior information. The media function, *g*, decreases as the exponentially-weighted moving average of the number of news articles increases, meaning that transmission is most slowed when there have been many articles published about the disease in the recent past. We used the exponentially-weighted moving average of news articles, assuming that articles published in the recent past would continue to have bearing on current behavior but that the level of influence would decrease with time. Let *θ*_*t*_ be the exponentially-weighted moving average of the number of news articles, with parameter λ ∈ (0, 1], controlling the relative weight of recent and prior information:

$$\begin{array}{c} \theta_{t}=\lambda (M_{t}+\left(1-\lambda \right)M_{t-1}+{\left(1-\lambda \right)}^{2}M_{t-2}+\cdots +{\left(1-\lambda \right)}^{t}M_{0}). \end{array}$$ (9)

Then we define the media function, *g*, as follows:

$$\begin{array}{c} g(M_{0},\ldots ,M_{t},\alpha ,\lambda )=e^{-\alpha \theta_{t}}. \end{array}$$ (10)

The parameter *α* > 0 determines the degree to which media reduces the per-contact transmission rate.

### Study design

We conducted several studies, including a sensitivity analysis of the model and several evaluations of the model in real-world scenarios. The first study explored the sensitivity of the model to variations in *α* and λ. We examined both the changes to the media function and to the resulting epidemic curve. In the second study, we incorporated real media coverage data into a model of 2009 A(H1N1) in Mexico City. We then compared the fitted media function with proposed approximate media functions, showing that, for this outbreak, approximate media functions cannot replicate the observed transmission dynamics. The final study demonstrated how the model could be used for analysis of a more typical disease outbreak. Simulations were fit to data from the 2014-2015 influenza season in Washington, DC. We compared the reduction in cases resulting from the observed level of media coverage with that expected from having no media coverage or increasing it ten times.

#### Parameter sensitivity analysis

Simulations were conducted on a network of 500,000 individuals (scale-free network with mean degree of 4), with 10 initially infected individuals. For baseline per-contact infection probability, *p*_0_ = 0.35, and time to recovery, *T*_*R*_ = 1, we simulated outbreaks varying the media parameters, *α* ∈ {0.0, 0.005, 0.01} and λ ∈ {0.1, 0.2, 1.0}. One week was used as the value of *T*_*R*_, since influenza cases typically recover within a week. The value of *p*_0_ was selected to generate an outbreak affecting a large portion of the population. Varying the values of *p*_0_ and *T*_*R*_ does not affect the interpretation of the roles of *α* and λ. Simulations were conducted on a network of 500,000 individuals (scale-free network with mean degree of 4), with 10 initially infected individuals. We then examined the effects of the parameters, *α* and λ, on the shape of the media function and the disease spread. For these simulations, there were five weeks without media, followed by five weeks with 100 articles each. The remaining weeks had no news articles published.

#### Mexico City

In 2009, there were two major outbreaks of H1N1 in Mexico City. The first began in mid-April [26]. The outbreak was relatively small and was controlled quickly via social distancing and a public information campaign [27]. The second outbreak began in August and spread much more widely than the first outbreak. The spring outbreak was met with intense media interest, while the fall outbreak received relatively little coverage. As there is no reason to believe that there were meaningful changes in the infectivity of the H1N1 virus or the social structure of Mexco City between the spring and fall outbreaks, the differences between the outbreaks needed to be explained by differences in media coverage.

In the model, the spring and fall outbreaks were simulated with the same parameters (i.e. *p*_0_, *T*_*R*_, *α* and λ) and the network structure was unchanged between simulations. Individuals who were infected during the spring outbreak were transferred to the recovered state prior to beginning the simulation of the fall outbreak, since they would have been immune to the virus. To model Mexico City we used a scale-free network [28] with mean degree 2*k* and 885,108 nodes [29], each representing 10 people.

The quality of fit was determined by mean absolute error (MAE) between the observed total number of cases and the median number of simulated cases per week taken over 1000 replications of the model, with the spring and fall outbreaks weighted equally. The MAE was weighted in order to prevent the fall outbreak, which lasted much longer, from being fit at the expense of the spring outbreak:

$$\begin{array}{c} MAE_{\text{weighted}}=(MAE_{\text{spring}}+MAE_{\text{fall}})/2. \end{array}$$ (11)

A greedy search of the parameter space, with random restarts, was implemented in order to determine the parameters that minimized the MAE.

#### Washington, DC

We fit data from the 2014-2015 influenza season in Washington, DC in order to evaluate the model on a typical seasonal disease outbreak. As with the Mexico City simulations, the quality of fit was determined by mean absolute error (MAE) between the observed total number of cases and the median number of simulated cases per week taken over 1000 replications of the model. Using the best-fit parameters, we considered the effects of having no media coverage or ten times more media coverage on the the spread of disease. By doing so, we could obtain an estimate of the role of media in limiting the spread of the disease. Simulations were to scale, with 658,893 individuals [30] reflecting the Washington, DC population, and were conducted on a scale-free network [28] with mean degree 2*k*.

### Data

For the Mexico City and Washington, DC studies, we collected data on the spread of disease, as well as the volume of media coverage and the availability of vaccines.

#### Mexico City

Data on number of H1N1 influenza cases per week were collected by the Mexican Social Security Institute [26]. H1N1 vaccines were not available until late November, 2009—too late to have had a large effect on the spread of disease in Mexico. Therefore, we did not consider vaccination in our analysis.

For influenza, reported confirmed cases represent only a small fraction of total cases. Therefore, it was necessary to scale the confirmed influenza cases. Seroprevalence studies, which estimate the percentage of the population with antibodies against the disease, are rare, and none is available for Mexico City. The best estimate of seroprevalence comes from a study conducted in Monterrey, Mexico. The researchers found that 33% of the general population of Monterrey had been infected [31]. We assumed that the disease prevalence in Mexico City was also 33%, and scaled the confirmed cases appropriately. We note, however, that there is substantial uncertainty surrounding the true size of the outbreak.

News articles published online which specifically reference the H1N1 influenza outbreak in Mexico City were collected by HealthMap, an Internet-based biosurveillance company [32]. The spring outbreak of H1N1 in Mexico City attracted intense media interest. There were 815 breaking news articles collected by HealthMap prior to June 1—about 20 articles per day from April 22 to June 1. News coverage of the fall outbreak was much more limited. Between September 17 and December 6, 66 published articles were collected by HealthMap—less than 1 article per day.

#### Washington, DC

The number of new influenza type A hospitalizations per week for the 2014-2015 influenza season was obtained from the Washington, DC Department of Health [33]. There were 615 hospitalizations during the season, which is typical for seasonal influenza in Washington, DC. The Washington, DC hospitalized cases were scaled to reflect total cases, assuming a hospitalization rate equal to the US hospitalization rate during the 2009-2010 H1N1 epidemic [34]. Data on the number of vaccinations per month was gathered from the US CDC [35]. The number of vaccinations during each week was estimated by assuming that all days within the month had the same number of vaccinations. The 2014-2015 influenza vaccine had low effectiveness, only 18% [36]. News articles about influenza in HHS Region 3—the region containing Washington, DC—were collected by HealthMap. Coverage of the outbreak was limited. The number of news articles per week peaked at the height of the influenza season, with 25 articles collected for the week ending January 1, 2015.

## Results

### Parameter sensitivity analysis

**Fig 1:**
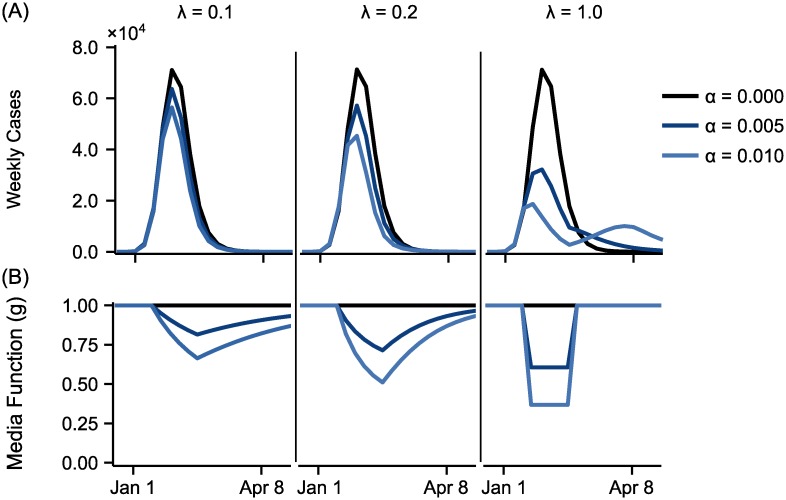
The effects of media function parameters. The effects of the media parameters, λ and *α*, on (A) the median number of cases per week and (B) the media function, *g*, are shown. As λ increases, the effect of media influence becomes more prominent but for a shorter duration. As *α* increases, media causes greater reductions in the rate of transmission.

In our proposed formulation, media exposure causes individuals to implement protective behaviors, reducing the per-contact transmission probability. Two parameters, *α* and λ, determine how the media signal affects the rate of transmission. Specifically, λ determines the relative importance of recent and prior news coverage in determining individual behavior. As λ increases, the relative weight of recent news articles increases, compared with prior news articles. The parameter *α* controls the degree to which media affects behavior. As *α* increases, media exerts greater influence on behavior, leading to greater reduction in the per contact transmission probability. The effects of λ and *α* on the number of cases and the media function *g* are shown in Fig 1 for an outbreak in a population of size 500,000 with five weeks of constant media coverage beginning five weeks after the beginning of the outbreak. As λ increases, the effect of media becomes more prominent but for a shorter duration. As λ increases, peak height of media function increases (for *α* greater than 0), indicating that the effect of media gets stronger (lower transmission probability). On the other hand, the rate at which the media function value goes back to one (no media effect) from the peak (maximum media effect) also increases as λ increases, suggesting a shorter duration of media effect. This phenomenon is explained by exponentially-weighted moving average function describing λ: smaller λ considers the media coverage from further back in the past, creating the lingering media effect as observed in the long tail of the media function curve for λ = 1. High values of λ can produce two wave outbreaks, if the value of *α* is high enough to dramatically slow transmission but not high enough to altogether stop transmission for long enough for the outbreak to die out. As *α* increases, media causes greater reductions in the rate of transmission.

### Case study: Mexico City

#### Best fit simulations

**Fig 2:**
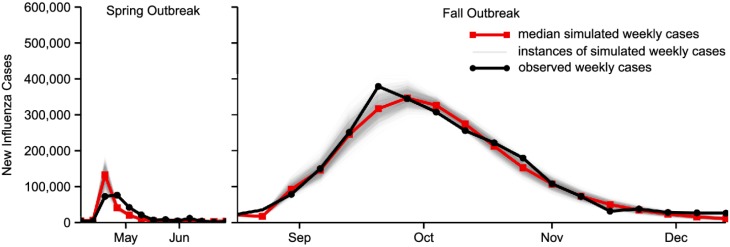
Simulation fit for 2009 A(H1N1) in Mexico City. Individuals took protective action after receiving a signal from the media that the disease was spreading. This protective action alone was sufficient to control the disease spread in the spring outbreak, accurately replicating the overall dynamics of the disease in Mexico City.

We fit the simulated epidemic curve from our model to the spring and fall outbreaks of H1N1 in Mexico City. An excellent overall fit was achieved, with a weighted mean absolute error of 1243 cases. In particular, the protective action implemented as a result of the media surge during the spring outbreak was sufficient to slow the outbreak. The fall outbreak, in contrast, spread nearly uninhibited, without generating extensive media attention. Fig 2 shows the simulation fit to the observed weekly influenza cases in Mexico City.

The best-fit parameters are listed in Table 1. The best-fit value of λ = 0.15 indicates that prior information about the disease decayed slowly. The largest value of *θ*_*t*_ was 92.5, and was achieved during the week starting April 26. During the week of April 26, the baseline per-contact infection probability was reduced by 89%. During the spring outbreak (April 5 through July 5, 2009), the average reduction in the baseline per-contact infection probability, resulting from media influence, was 62.4%. During the fall outbreak, the average reduction was 17.0%.

**Table 1:** Best-fit parameters for Mexico City 2009 A(H1N1). All parameters were fit to data.

Parameter	Description	Value
*p*_0_	Baseline per-contact infection probability	0.336
*k*	$\frac{1}{2}$ the mean degree of the network	2
*α*	Effect of media on protective behavior	0.024
λ	Relative weight of recent information compared with prior information	0.15

#### Comparing model performance using different media functions and not using a media function

The most common media function from the literature takes the form:

$$\begin{array}{c} f(I,\frac{dI}{dt},a,b)=e^{-\max\{0,\;aI+b\frac{dI}{dt}\}}. \end{array}$$ (12)

**Fig 3:**
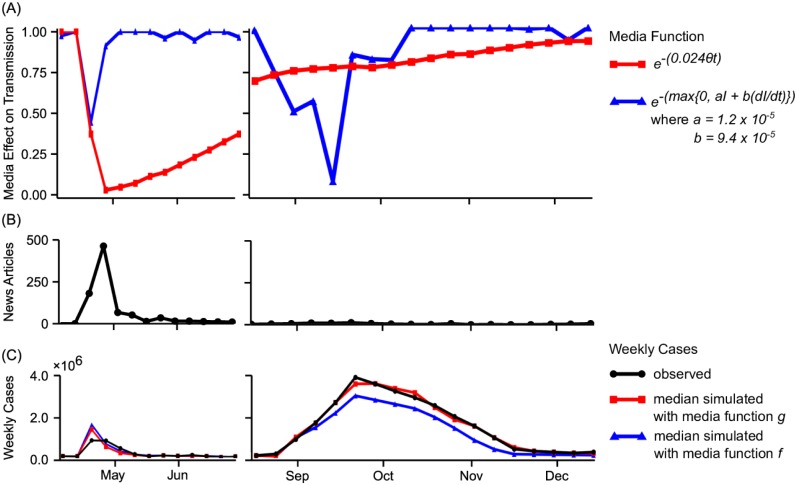
Comparison of media functions and simulated best-fit curves for 2009 A(H1N1) in Mexico City. (A) The proposed media function, *g*, based on the number of published news articles (red line with square markers) is compared with the media function, *f*, based on the number of infected individuals and the change in the number of infected individuals (blue line with triangular markers). (B) The number of news articles per week is depicted. (C) The best-fit curve using the proposed media function is compared with the best-fit curve using the media function *f*. The proposed media function accurately captures both the large reduction in the spread of disease during the spring outbreak, due to social distancing, and the insignificant reduction during the fall outbreak. Media functions based on the number of cases and rate of spread reduce the spread of disease more substantially during the fall than during the spring, largely underestimating the fall outbreak.

In this formulation, the transition rate from the susceptible to infected disease states decreases with *f*. When *a* = 0 and *b* > 0, increases in the rate of change of the infected population lead to reductions in the transmission rate. When *a* > 0 and *b* = 0, increases in the size of the infected population lead to reductions in the transmission rate. When *a* > 0 and *b* > 0, both the size of the infected population and the rate of change affect the transmission rate. Fig 3A compares the proposed media function, *g*, with the media function, *f*, each using its best-fit parameters for Mexico City. Function *f* was fit using the same implementation, the best-fit parameters values equal to *p*_0_ = 0.319, *k* = 2, *a* = 1.2 × 10^−5^, and *b* = 9.4 × 10^−5^. The actual number of news articles per week and the observed number of new cases per week are shown in Fig 3B and 3C. Fig 3C compares the best-fit curve obtained using the proposed media function with the best-fit curve obtained using the media function *f*.

Since *f* is dependent upon the rate of change and size of the infected population, *f* results in the greatest reduction in transmission during the fall outbreak, instead of the spring outbreak. As depicted in Fig 3C, *f* overestimates the fall outbreak and underestimates the spring outbreak, resulting in much greater mean absolute error of 3733, three times the MAE obtained using the proposed media function. We have also compared the best-fit curve obtained using the proposed media function, *g*, with the best-fit curve obtained without using a media function to evaluate the effectiveness of the media function. The best-fit parameters were *p*_0_ = 0.289 and *k* = 2. The model without a media function overestimates both the spring and fall outbreaks, resulting in MAE of 1865, fifty percent over the MAE obtained using the proposed media function. During the spring outbreak where significant media coverage was observed, the model overestimates the peak by over twice as much. In order to replicate the dynamics of the two outbreaks of disease without considering additional control measures, consideration of factors affecting behavior other than the number of infected cases or rate of change in the number of infected cases is necessary. We found that use of actual media data fills the void, allowing us to account for the heightened attention to the disease in the spring that led to the extreme social distancing that eventually curtailed the outbreak.

The proposed model relies on volume of news coverage to estimate the media signal. We have compared the results of modeling disease spread dynamics over time using real data on news coverage as a proxy of media signal versus that utilizing the number of cases and their temporal change and that not using any media signal. We have demonstrated a significantly better model fit when utilizing the proposed media signal.

### Case study: Washington, DC

**Fig 4:**
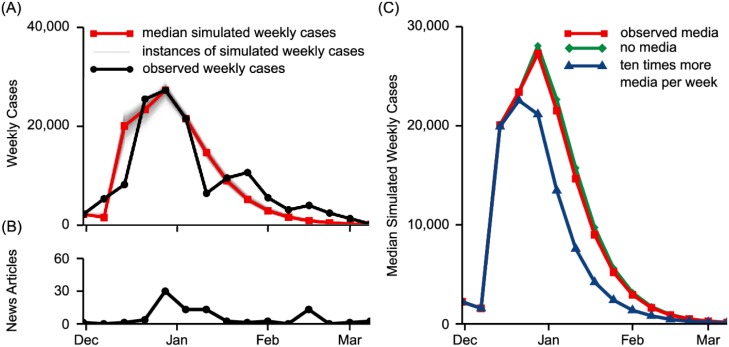
Simulated epidemic curves for 2014-2015 influenza season in Washington, DC. (A) The best-fit parameters achieved an excellent fit to the observed cases, with a mean absolute error of 2818 cases. (B) Few news articles were published about influenza in HHS Region 3, during the 2014-2015 influenza season. (C) Using the best-fit parameters, we simulated outbreaks with varying levels of media coverage. The median number of cases in simulations conducted with the observed level of media coverage was not substantially different than the median number in simulations conducted assuming no media coverage. Increasing the number of news articles per week by ten times, resulted in a 33.5% decrease in the median number of cases, compared with the simulations conducted with the observed number of news articles per week.

We fit our model to the epidemic curve from the 2014-2015 influenza season in Washington, DC. The resulting fit had a MAE of 2818 cases. Our simulations indicate that media had limited effect on behavior during the 2014-2015 influenza season in Washington, DC, due to the small number of news articles published. In the best-fit simulations, the effect of each news article on protective behavior was moderate (*α* = 0.004, Table 2), but the overall effect of media was small. Excluding the effects of media resulted in only a 3.5% increase in the median number of cases (Fig 4). When the number of news articles per week was increased ten-fold in our simulations, media played a greater role, with a 33.5% decrease in the median number of cases.

**Table 2:** Best-fit parameters for 2014-2015 influenza in Washington, DC. All parameters were fit to data.

Parameter	Description	Value
*p*_0_	Baseline per-contact infection probability	0.199
*k*	$\frac{1}{2}$ the mean degree of the network	2
*α*	Effect of media on protective behavior	0.004
λ	Relative weight of recent information compared with prior information	0.23

## Discussion

Previous models of infectious disease transmission that incorporate media influence have modeled media as a function of the rate of transmission and the number of cases of a disease, making it dependent upon the size of the outbreak. We have shown that media signal can be successfully modeled using real data on news coverage, and that using actual news coverage data better captures the disease dynamics compared to the estimation using the media function that uses the number of cases and rate of change in the number of cases.

First, we demonstrated the theoretical properties of the proposed media function. The media function is affected by the observed volume of media coverage, as well as two parameters, λ and *α*, with λ controlling the relative weight of recent and prior information in determining behavior and *α* controlling the extent to which transmission is slowed by media exposure. As λ increases, the effect of media is more prominent and has shorter duration. As *α* increases, the spread of disease is slowed more quickly, though there can be multiple waves of infection when the values of *α* and λ are both relatively high.

Secondly, we provided a real world example that illustrates where models incorporating data on actual media coverage can be most useful. Although there were no significant differences in the per-contact transmission rate of the H1N1 virus or population structure, the spring outbreak of H1N1 in Mexico City was quickly contained, while the fall outbreak grew to epidemic proportions. Differences in social distancing help to explain the different dynamics of the two outbreaks. The spring outbreak received extensive attention in the Mexican press. By incorporating data on the volume of media coverage of the outbreak and by assuming that media influence leads to increased protective practices and therefore reduction in the per-contact transmission probability, we were able to account for the social distancing that took place during the spring outbreak but which did not occur during the fall outbreak, achieving a good overall fit to the epidemic curves for the spring and fall outbreaks using the same model for both outbreaks. The Mexico City H1N1 example shows that there is not always a the direct link between case counts and media attention. Therefore, it is important to quantify actual media attention in infectious disease models.

Public reaction to common or seasonal diseases can be limited [14], even though such diseases are often deadly. Using data from the 2014-2015 influenza season in Washington, DC, we explored the role of media in a typical, seasonal outbreak and demonstrated that the low level media interest exhibited during the influenza season was associated with only a small reduction in the number of cases. Media may be a tool for public health officials to communicate preventive measures to the public during disease outbreaks. Therefore, the effect of significantly increasing the volume of media coverage was explored. It was found that a ten-fold increase in the volume of media coverage resulted in a 33.5% decrease in the median number of infections. Thus, media may be a moderately effective means to prevent disease transmission.

In conclusion, we have proposed a data-driven approach to incorporating the effect of media into models of infectious disease transmission, and have illustrated the effectiveness of the approach by fitting data from recent disease outbreaks. We believe media reaction can serve as a good proxy for the population reaction to disease outbreaks, and is substantially easier to measure accurately and quickly. Thus, as our study illustrates, incorporating media coverage allows for more accurate modeling of infectious disease outbreaks during which there are substantial changes in population behavior.
